# Elimination of Image Saturation Effects on Multifractal Statistics Using the 2D WTMM Method

**DOI:** 10.3389/fphys.2022.921869

**Published:** 2022-06-28

**Authors:** Jeremy Juybari, Andre Khalil

**Affiliations:** ^1^ CompuMAINE Lab, University of Maine, Orono, ME, United States; ^2^ Department of Electrical and Computer Engineering, University of Maine, Orono, ME, United States; ^3^ Department of Mathematics and Statistics, University of Maine, Orono, ME, United States; ^4^ Department of Chemical and Biomedical Engineering, University of Maine, Orono, ME, United States

**Keywords:** multifractal, wavelets, image saturation, mammography, breast cancer

## Abstract

Imaging artifacts such as image saturation can restrict the computational analysis of medical images. Multifractal analyses are typically restricted to self-affine, everywhere singular, surfaces. Image saturation regions in these rough surfaces rob them of these core properties, and their exclusion decreases the statistical power of clinical analyses. By adapting the powerful 2D Wavelet Transform Modulus Maxima (WTMM) multifractal method, we developed a strategy where the image can be partitioned according to its localized response to saturated regions. By eliminating the contribution from those saturated regions to the partition function calculations, we show that the estimation of the multifractal statistics can be correctly calculated even with image saturation levels up to 20% (where 20% is the number of saturated pixels over the total number of pixels in the image).

## 1 Introduction

Instrumentation noise, hardware failures, and inadequate camera or spectrometer calibration can lead to imaging artifacts (Walz-Flannigan et al., 2018; Liu H. et al., 2021). In many circumstances, a quality control procedure eliminates imaging data that do not pass a minimal quality requirement and the samples are re-imaged. However, in certain situations, in particular for imaging data obtained from human samples (e.g., screening mammography), re-imaging is often much more difficult, if not impossible. In other situations, images containing artifacts may still be usable for subjective analyses via visual inspection, but would otherwise be inadequate for objective, computational image analysis pipelines. Therefore, efforts are needed to palliate the deficiencies caused by imaging artifacts on sensitive computational analyses.

**FIGURE 1:**
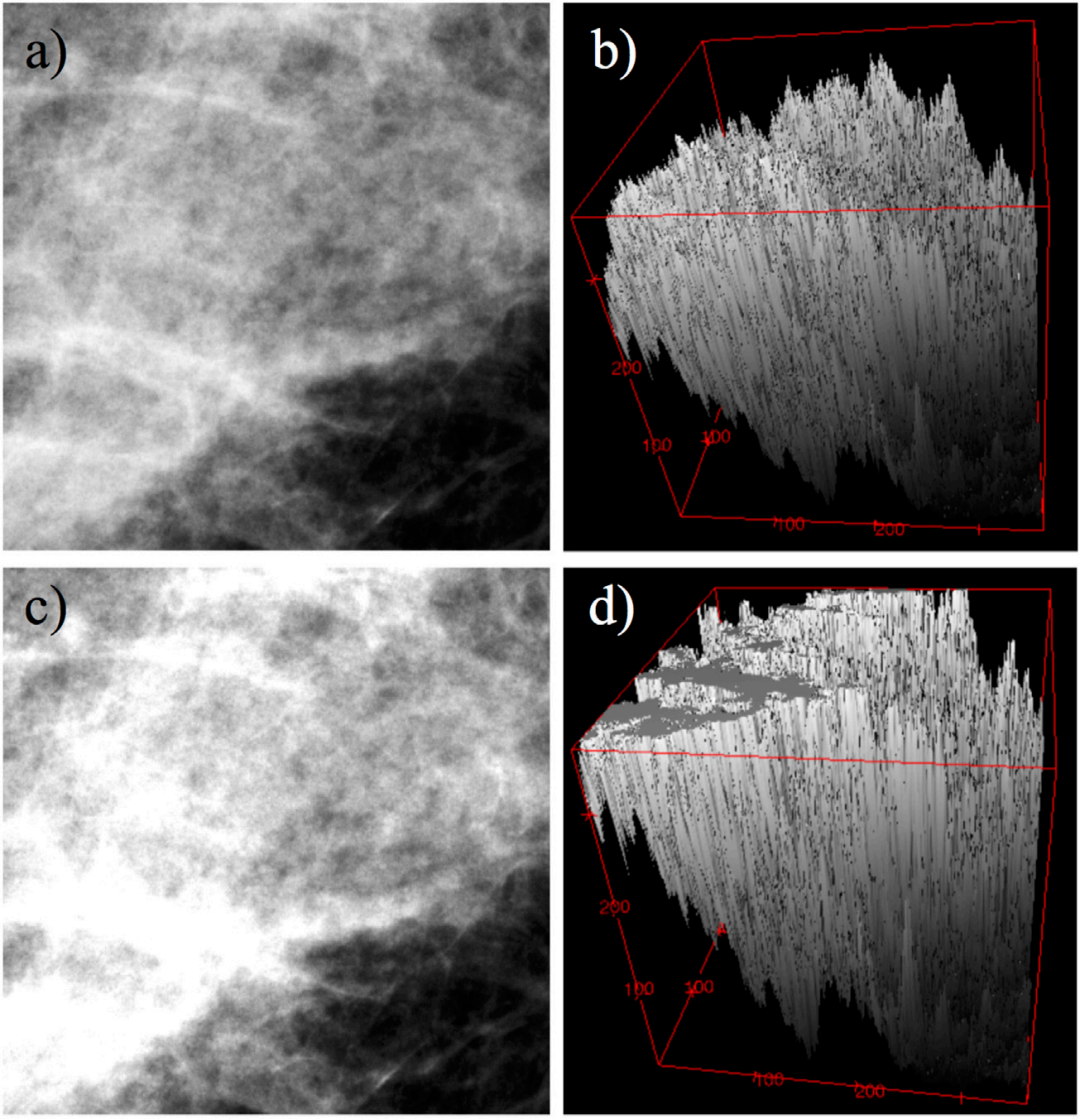
Example of saturation effects on a 360, ×, 360 pixel subregion from a mammogram. **(A)** Original image. **(B)** Mesh representation of image **(A)** as a rough surface. **(C)** Same image as in **(A)**, but saturated at 20%. **(D)** Mesh representation of image **(C)** as a rough surface.

Image saturation is a type of distortion where a portion of the acquired image is limited to some maximal pixel value (Figure 1). It can cause problems for computer vision algorithms that assume linearity, unless saturated pixels are identified and handled appropriately (Hasinoff, 2014). Typical approaches to deal with saturated pixels are to either ignore them (eliminate from the analysis) or interpolate their values based on neighboring pixels (Masood et al., 2009). Image saturation also affects calculations in the Fourier domain and needs a strategy to be mitigated (Wetzstein et al., 2010).

The multifractal analysis of self-affine rough surfaces (Arneodo et al., 2000; Decoster et al., 2000; Arneodo et al., 2003; Wendt et al., 2009) is very valuable in many applications with underlying multiscale non-linear variability, from astrophysics (Khalil et al., 2006; Kestener et al., 2010), geophysics (Roux et al., 2000), texture-based segmentation (Pascal et al., 2018), to biomedical imaging (Kestener et al., 2001; Marin et al., 2017; Gerasimova-Chechkina et al., 2021).

Saturation can easily perturb the multifractal analysis, especially for negative statistical order moments, as discussed in detail below. Indeed, any method that uses either an increment or gradient-based approach to estimate the multifractal signature of rough surfaces (Arneodo et al., 2000; Decoster et al., 2000; Arneodo et al., 2003; Khalil et al., 2006) risks being affected by regions of saturation within the image. Mathematically-speaking, such artifactual images do not possess truly self-affine properties (e.g., everywhere continuous but nowhere differentiable) because these saturation regions are flat and thus infinitely differentiable. Therefore, any estimation of multifractal statistics from such images would be incorrect. To the best of our knowledge, no approach exists in the current literature to rescue the effects of image saturation on multifractal statistics.

The 2D Wavelet Transform Modulus Maxima (WTMM) method has been adapted and applied in three different forms: a multiscale segmentation method (Kestener et al., 2001; Khalil et al., 2007; Roland et al., 2009; Grant et al., 2010; Kestener et al., 2010; McAteer et al., 2010; Batchelder et al., 2014; Marin et al., 2018; Liu J. et al., 2021), a multiscale anisotropy method (Khalil et al., 2006, 2009; Tilbury et al., 2021), and a multifractal formalism (Arneodo et al., 2000; Decoster et al., 2000; Roux et al., 2000; Arneodo et al., 2003; Khalil et al., 2006). The 2D WTMM multifractal method is a multiscale formalism perfectly suited for the analysis of self-affine rough surfaces such as mammograms by identifying density fluctuations and spatial correlations within these surfaces (Batchelder et al., 2014; Plourde et al., 2016; Marin et al., 2017; Gerasimova-Chechkina et al., 2021).

In this manuscript, we demonstrate how the 2D WTMM multifractal method, thanks to the ease with which its space-scale skeleton can be partitioned, allows one to eliminate the effects of saturation on the multifractal statistics. Moreover, we show that the implementation of the strategy used to eliminate these effects does not preclude the analysis of normal (non-saturated) images. In Section 2, a detailed recapitulation of the 2D WTMM multifractal method is presented. The effects of image saturation on the calculation of the multifractal statistics and the rescue method are presented in Section 3, followed by the conclusion and discussion in Section 4.

## 2 Materials and Methods

### 2.1 The 2D WTMM Multifractal Method

Let *f* be a function from $R^{2}$ into R and *S*_*h*_ the set of all points *x*_0_ so that the Hölder exponent of *f* at *x*_0_ is *h*. The singularity spectrum *D*(*h*) of *f* is the function which associates with any *h*, the fractal dimension of *S*_*h*_

$$D\left(h\right)=D_{F}\left\{x\in R^{2}\text{ , }h\left(x\right)=h\right\}.$$

The continuous wavelet transform is defined as

$$T_{\psi }\left[f\right]\left(b,a\right)=\left(\begin{array}{c} T_{\psi_{1}}\left[f\right]=a^{-2}\int \psi_{1}\left(a^{-1}\left(x-b\right)\right)f\left(x\right)d^{2}x\\ T_{\psi_{2}}\left[f\right]=a^{-2}\int \psi_{2}\left(a^{-1}\left(x-b\right)\right)f\left(x\right)d^{2}x \end{array}\right)$$

where **b** is the space parameter, $f\in L^{2}\left(R\right)$ , *a* represents scale, **x** = (*x*, *y*), and where the first-order wavelets are defined as (Arneodo et al., 2000)

$$\psi_{1}\left(x,y\right)=\frac{\partial G\left(x,y\right)}{\partial x}\text{ and }\psi_{2}\left(x,y\right)=\frac{\partial G\left(x,y\right)}{\partial y}$$

with

$$G\left(x,y\right)=e^{-\left(x^{2}+y^{2}\right)/2}=e^{-|x{|}^{2}/2}.$$

The wavelet transform can be expressed in terms of its modulus $M_{\psi }\left[f\right]\left(b,a\right)$ and argument $A_{\psi }\left[f\right]\left(b,a\right)$ :

$$T_{\psi }\left[f\right]\left(b,a\right)=\left(M_{\psi }\left[f\right]\left(b,a\right),A_{\psi }\left[f\right]\left(b,a\right)\right),$$ (1)

where

$$M_{\psi }\left[f\right]\left(b,a\right)=\sqrt{{\left(T_{\psi_{1}}\left[f\right]\left(b,a\right)\right)}^{2}+{\left(T_{\psi_{2}}\left[f\right]\left(b,a\right)\right)}^{2}}$$

$$A_{\psi }\left[f\right]\left(b,a\right)=Arg\left(T_{\psi_{1}}\left[f\right]\left(b,a\right)+iT_{\psi_{2}}\left[f\right]\left(b,a\right)\right).$$

**FIGURE 2:**
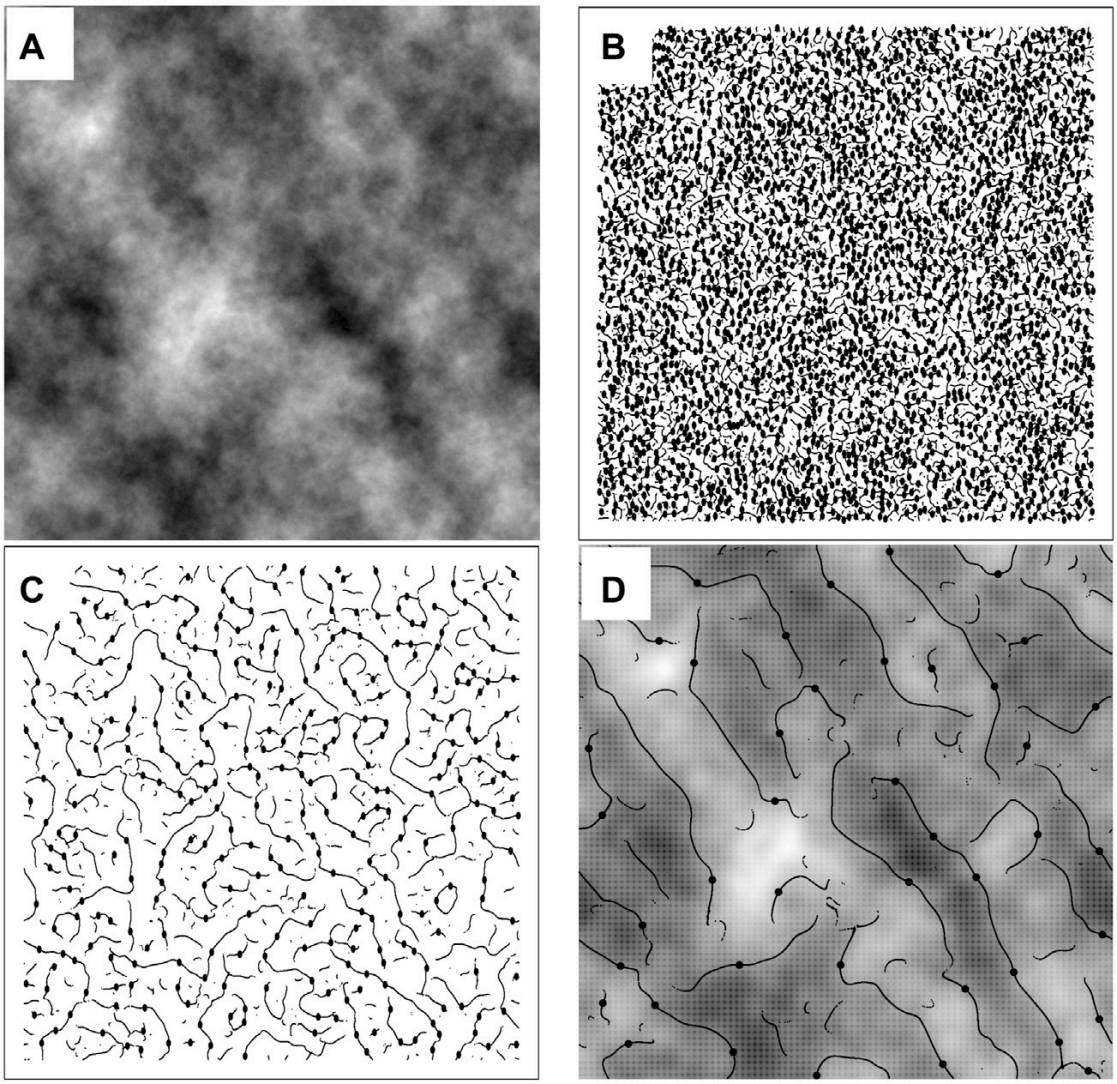
**(A)** A 512 × 512-pixel fBm surface with *H* = 0.7. The maxima chains, i.e., positions where the modulus $M_{\psi }\left[f\right]\left(b,a\right)$ from Eq. 1 is maximum, are shown as black lines while the WTMMM are represented as black dots for scale *a* = 20 pixels **(B)**, *a* = 56 pixels **(C)**, and *a* = 158 pixels **(D)**. Also shown in **(D)** is the overlay of the maxima chains and WTMMM on the original image smoothed with the Gaussian function G at scale *a* = 158 pixels. The WTMMM are then connected across scales to make the WT Skeleton shown in Figure 3.

The Wavelet Transform Modulus Maxima (WTMM) are locations **b** where $M_{\psi }\left[f\right]\left(b,a\right)$ is a local maximum in the angular direction of $A_{\psi }\left[f\right]\left(b,a\right)$ for a given scale *a*. The WTMM capture the gradient changes in the underlying rough surface. The WTMM are on connected chains, called maxima chains (Arneodo et al., 2000). This process is repeated at every scale. An example for a 2D fractional Brownian motion (fBm) surface (Mandelbrot and Ness, 1968) with Hurst roughness exponent *H* = 0.7 is provided in Figure 2A, where the black edge detection lines shown in Figures 2B–D are the WTMM chains.

**FIGURE 3:**
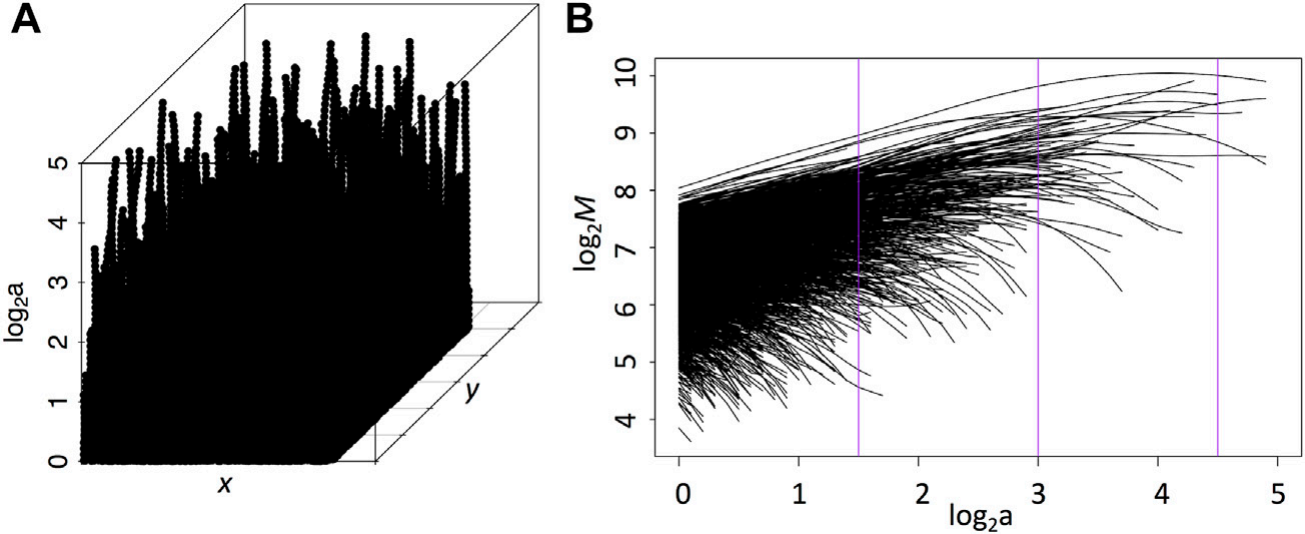
**(A)** WT Skeleton: each vertical line connecting the WTMMM through the scales, by linking the WTMMM closest at the next scale, is known as a maxima line. Each WTMMM point along these maxima lines have a modulus value at each (*x*, *y*, log_2_(*a*)) position. **(B)** Sheaf: One can study the behavior of the modulus as a function of the scale parameter for each maxima line by eliminating the positional information (see Eq. 3). The three vertical purple lines correspond to scales 20, 56, 158 pixels represented in Figures 2B–D. In these two plots, the scale parameter is expressed in units of 7*2^*a*^ pixels.

The WTMM maxima (WTMMM) are defined as the points along the maxima chains where $M_{\psi }\left[f\right]\left(b,a\right)$ is locally maximum (black dots in Figures 2B–D). These WTMMM are connected through scales, *a*, and form individual maxima lines. The set of all maxima lines is called the Wavelet Transform (WT) space-scale skeleton, as illustrated in Figure 3A.

Along a maxima line pointing to the singularity **x**_**0**_ in the rough surface as *a* → 0^+^, denoted $L_{x_{0}}\left(a\right)$ , the WTMMM follow (Mallat and Hwang, 1992; Arneodo et al., 2000)

$$M_{\psi }\left[f\right]\left(L_{x_{0}}\left(a\right)\right)∼a^{h\left(x_{0}\right)},a\to 0^{+}$$ (2)

where *h* (**x**_**0**_) is the Holder roughness exponent. Note that Eq. 2 only holds if the wavelet order (= 1) is greater than the Holder exponent (Mallat and Hwang, 1992; Arneodo et al., 2000), which is a safe assumption to make in this study since all surfaces considered here have roughness exponents less than 1.

This means that *h* (**x**_**0**_) can be estimated by considering

$$\frac{\log_{2}\left(M_{\psi }\left[f\right]\left(L_{x_{0}}\left(a\right)\right)\right)}{\log_{2}\left(a\right)}∼h\left(x_{0}\right).$$ (3)

Figure 3B shows such a log-log plot for all of the maxima lines shown in Figure 3A. This is called a sheaf.

### 2.2 Partition Function and Statistical Order Moments

Let L(a) be the set of maxima lines at scale *a* and define the partition functions as (Arneodo et al., 2000, 2003; Khalil et al., 2006):

$$Z\left(q,a\right)=\underset{l\in L\left(a\right)}{\sum }{\left(\underset{\left(b,a^{\prime }\right)\in l,a^{\prime }\leq a}{\sup}M_{\psi }\left[f\right]\left(b,a^{\prime }\right)\right)}^{q}$$ (4)

where *q* are the statistical order moments. Note that negative *q* values give more weight to small modulus values while positive *q* values give more weight to large modulus values. The following power-law relationship allows us to estimate the roughness of a surface (see Arneodo et al. (2003) and references therein):

$$Z\left(q,a\right)∼a^{\tau \left(q\right)},a\to 0^{+}.$$ (5)

For a monofractal rough surface such as a fBm surface, this *τ*(*q*) spectrum is a linear function of *q* where the slope of *τ*(*q*) gives an estimate for the Hurst exponent, *H*, i.e., (Arneodo et al., 2000):

$$\tau \left(q\right)=qH-2.$$ (6)

However for multifractal rough surfaces, *τ*(*q*) is nonlinear which highlights the varying roughness exponents in the underlying surface (Decoster et al., 2000).

A Legendre transform can be applied to the *τ*(*q*) spectrum to obtain the *D*(*h*) spectrum of singularities (Arneodo et al., 2000, 2003; Khalil et al., 2006):

$$D\left(h\right)=\underset{q}{\min}\left(qh-\tau \left(q\right)\right).$$ (7)

Given the numerical impediments related to the Legendre transform (Arneodo et al., 1995), one can avoid directly performing the Legendre transform by considering *h* and *D*(*h*) as mean quantities defined in a canonical ensemble, i.e., with respect to their Boltzmann weights computed from the WTMMM (Arneodo et al., 2000):

$$W_{\psi }\left[f\right]\left(q,l,a\right)=\frac{{\left|\sup_{\left(b,a^{\prime }\right)\in l,a^{\prime }\leq a}M_{\psi }\left[f\right]\left(b,a^{\prime }\right)\right|}^{q}}{Z\left(q,a\right)}$$

where *Z* (*q*, *a*) was defined in Eq. 4. One can then compute the expectation values:

$$h\left(q,a\right)=\underset{l\in L\left(a\right)}{\sum }\ln\left|\underset{\left(b,a^{\prime }\right)\in l,a^{\prime }\leq a}{\sup}M_{\psi }\left[f\right]\left(b,a^{\prime }\right)\right|W_{\psi }\left[f\right]\left(q,l,a\right)$$ (8)

and

$$D\left(q,a\right)=\underset{l\in L\left(a\right)}{\sum }W_{\psi }\left[f\right]\left(q,l,a\right)\ln W_{\psi }\left[f\right]\left(q,l,a\right).$$ (9)

**FIGURE 4:**
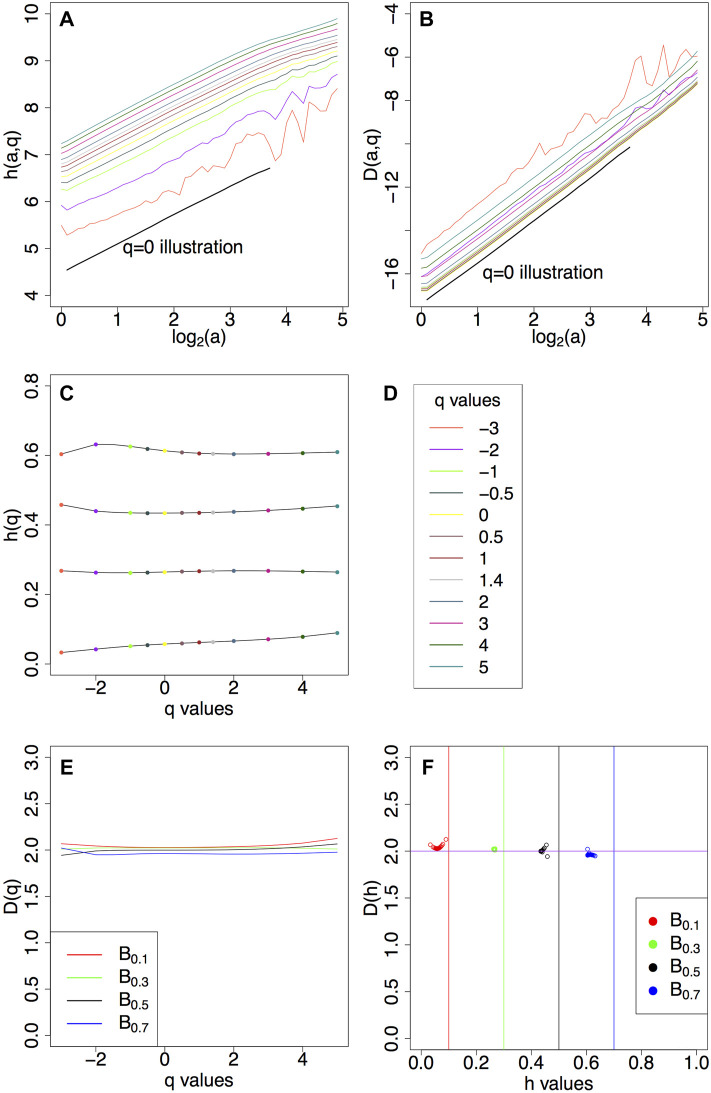
**(A)** *h* (*a*, *q*) curves (Eq. 8) and **(B)** *D* (*a*, *q*) curves (Eq. 9) for *q* values from −3 to 5 for fBm surfaces with *H* = 0.5. **(C)** *h*(*q*) (Eq. 10) for fBms with *H* = 0.1, 0.3, 0.5, 0.7. **(D)** List of *q* values used. Corresponding **(E)** *D*(*q*) (Eq. 11) and **(F)** *D*(*h*).

This gives the following:

$$h\left(q\right)=\frac{d\tau \left(q\right)}{dq}=\underset{a\to 0^{+}}{\lim}\frac{h\left(q,a\right)}{\ln a}$$ (10)

$$D\left(q\right)=\underset{a\to 0^{+}}{\lim}\frac{D\left(q,a\right)}{\ln a},$$ (11)

from which we obtain the *D*(*h*) singularity spectrum. Numerical calculations following the steps outlined above are presented in Figure 4 for sample fBm surfaces with *H* = 0.1, 0.3, 0.5, 0.7.

Statistical order moments (*q* values) play a crucial role in the 2D WTMM multifractal method. These values allow one to emphasize different singularity strengths of the underlying surface by weighing the modulus of the wavelet transform along the maxima lines. The quantity and quality of the underlying data will determine the range of the available statistical order moments (for an in-depth discussion, see Khalil et al., 2006).

### 2.3 Numerical Implementation

We followed previously established numerical calibration studies using the 2D WTMM multifractal method (Arneodo et al., 2000; Decoster et al., 2000; Khalil et al., 2006). For each Hurst value explored, 32 synthetic fBm surfaces 1,024 × 1,024 pixels in size were generated using a Fourier filtering synthesis algorithm (see Arneodo et al. (2000) and references therein). The results presented in this manuscript correspond to the average partition functions over these sets of 32 surfaces.

Saturation was introduced by considering the cumulative density distribution of pixel values for a given image and determining the critical pixel value corresponding to the saturation level desired. Then all pixel values above that critical value were changed to the maximal value.

The calculations of *h*(*q*) and *D*(*q*) were obtained from the slope of the linear fit of the log-log representation of the *h* (*q*, *a*) (Eq. 10) and *D* (*q*, *a*) (Eq. 11) curves, respectively. In this manuscript, a fixed range of scales was used for fBm surfaces of all *H* values and all saturation levels: from 17 to 56 pixels.

The numerical calculations described in this section were performed using Xsmurf, a Tcl\Tk software package that runs C-based routines (https://github.com/pkestene/xsmurf).

## 3 Results

### 3.1 Effects of Saturation on WTMM Statistics

**FIGURE 5:**
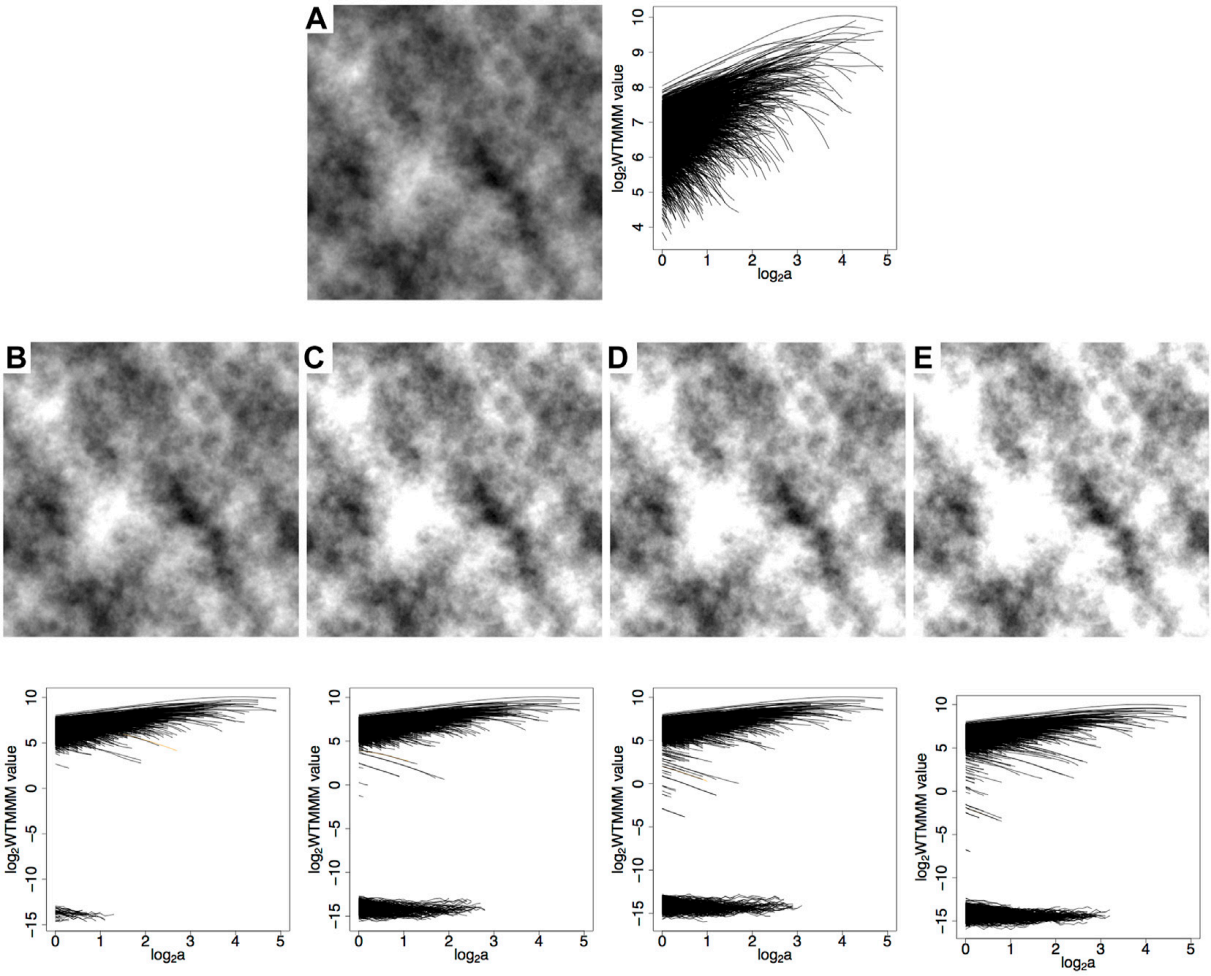
**(A)** A sample fBm surface with *H* = 0.7 and its corresponding sheaf (right). **(B)** The same fBm surface as in **(A)** but saturated at 1% and the corresponding sheaf directly below. **(C–E)** Saturated fBms at **(C)** 5%, **(D)** 10%, and **(E)** 20% and sheafs below. Maxima lines associated with extremely low modulus values, and others with an overall negative slope, appear in higher numbers with increasing saturation. These extraneous maxima lines will interfere with *h* (*a*, *q*) and *D* (*a*, *q*) calculations, which then reduces the number of statistical order moments as shown Figure 6.

The apparition of extraneous maxima lines in the sheafs of saturated fBm images are displayed in Figure 5. Compared to the original, non-saturated fBm surfaces, two categories of new maxima lines appear in higher numbers with increasing saturation: 1) maxima lines associated with extremely low modulus values, and 2) other maxima lines with an overall negative slope.

**FIGURE 6:**
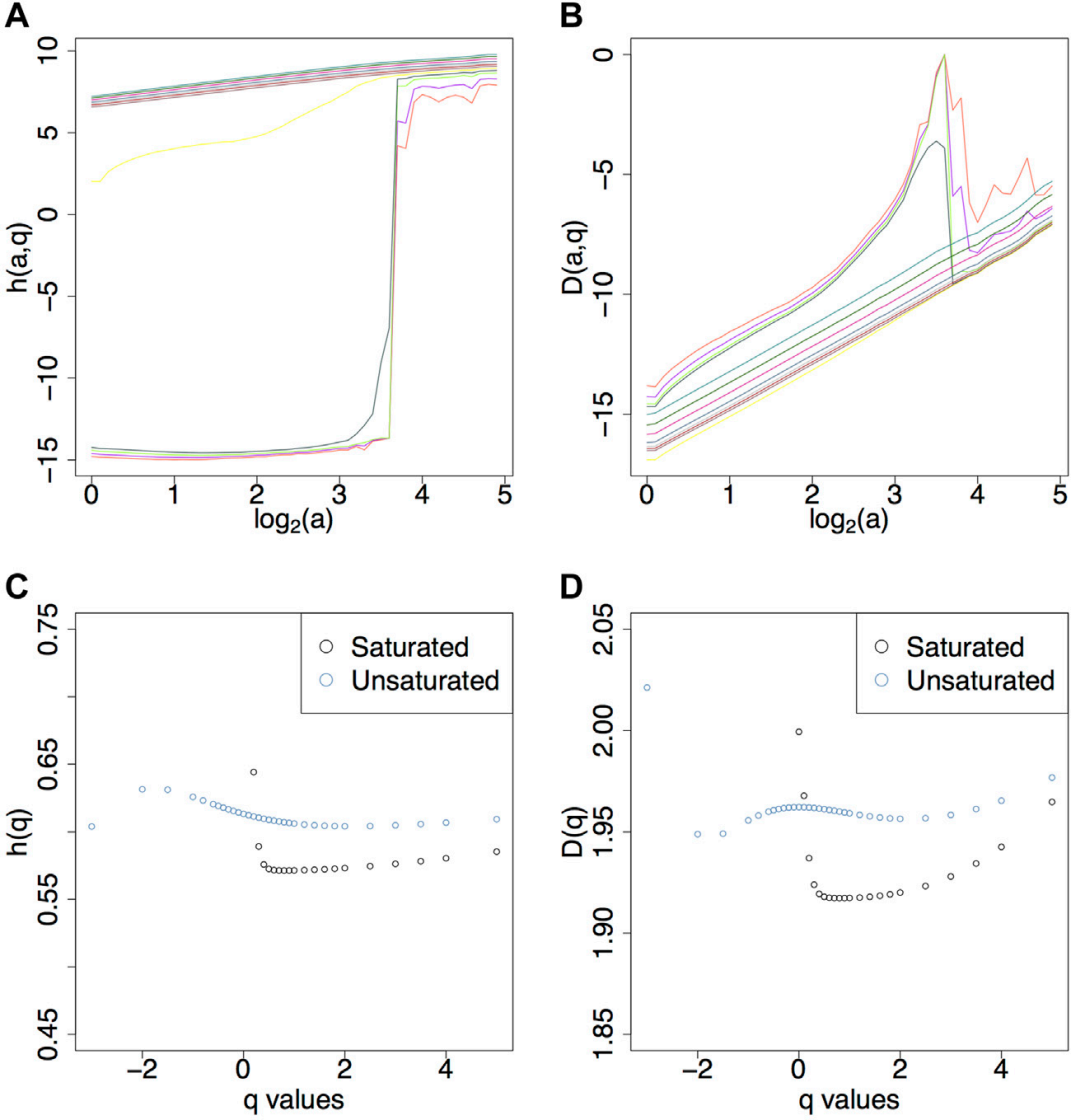
**(A)** *h* (*a*, *q*), **(B)** *D* (*a*, *q*), **(C)** *h*(*q*), and **(D)** *D*(*q*) for *H* = 0.7 with 20% saturation. The colors corresponds to the different *q* values as described in the legend in Figure 4D. Image saturation reduces the range of statistical order moments because of the lack of a good power law fit in the *h* (*a*, *q*) and *D* (*a*, *q*) plots. Thus in the corresponding *h*(*q*) and *D*(*q*) plots there are less statistics for saturated images (black dots). Similar plots are shown in the Supplementary Material for saturation levels 1, 5, and 10%.

Keeping these maxima lines in the skeleton that is fed to the partition function calculation (Eq. 4) affects the behavior of the *h* (*a*, *q*) and *D* (*a*, *q*) curves for a range of *q* values, as shown in Figures 6A,B. The non-linear behavior of log-log representation of the *h* (*a*, *q*) and *D* (*a*, *q*) curves for several *q* values precludes the calculation of *h*(*q*) (Eq. 10) and *D*(*q*) (Eq. 11), as shown in Figures 6C,D.

As a consequence, whereas a range of −3 ≤ *q* ≤ 5 was adequate for the non-saturated fBms (Figure 4), this range becomes limited to ∼0<q≤5 for the saturated fBms of *H* = 0.7, regardless of the saturation level (1, 5, 10, 20%).

### 3.2 The Rescue Method on Saturated Surfaces

We propose an approach to eliminate the two categories of extraneous maxima lines that are responsible for the bad power-law behavior of the *h* (*a*, *q*) and *D* (*a*, *q*) curves. As discussed below, this technique is referred to as the rescue method. In short, it consists of eliminating a subset of maxima lines by applying a first threshold filter on the modulus value at scale *a* = 1 (referred to below as *MF*), and a second adjusted slope threshold filter (referred to below as *SF*).

Let l∈L(a) be a maxima line ending at scale *a*_max_ and define $M_{l\left(a=a^{\prime }\right)}$ as the value of the modulus for maxima line *l* at scale *a*′. The modulus filter (*MF*) is used to eliminate the maxima lines that have a low modulus value at scale *a* = 1, i.e., when $M_{l\left(a=1\right)}\leq MF$ .

Next, we define the adjusted slope, *m*_*adj*_, as:

$$m_{\mathrm{adj}}=\frac{\log_{2}\left(M_{l\left(a=1\right)}\right)-\log_{2}\left(M_{l\left(a=a_{max}\right)}\right)}{\log_{2}\left(M_{l\left(a=a_{max}\right)}\right)}.$$

Therefore, to calculate *m*_*adj*_, one takes the starting modulus value of a maxima line (at scale *a* = 1) and subtracts the ending modulus value (at scale *a* = *a*_max_), all divided by the ending modulus value. For a given slope filter threshold, *SF*, the subset of maxima lines that satisfy *m*_*adj*_ ≥ *SF*, are eliminated.

### 3.2.1 Determination of Numerical Filter Parameters *SF* and *MF*

When comparing sheafs from saturated surfaces to sheafs from non-saturated surfaces, we empirically determined that any maxima lines with $M_{l\left(a=1\right)}$ of 16 (2^4^) or less should be removed, i.e., log_2_ (*MF*) = 4.

**FIGURE 7:**
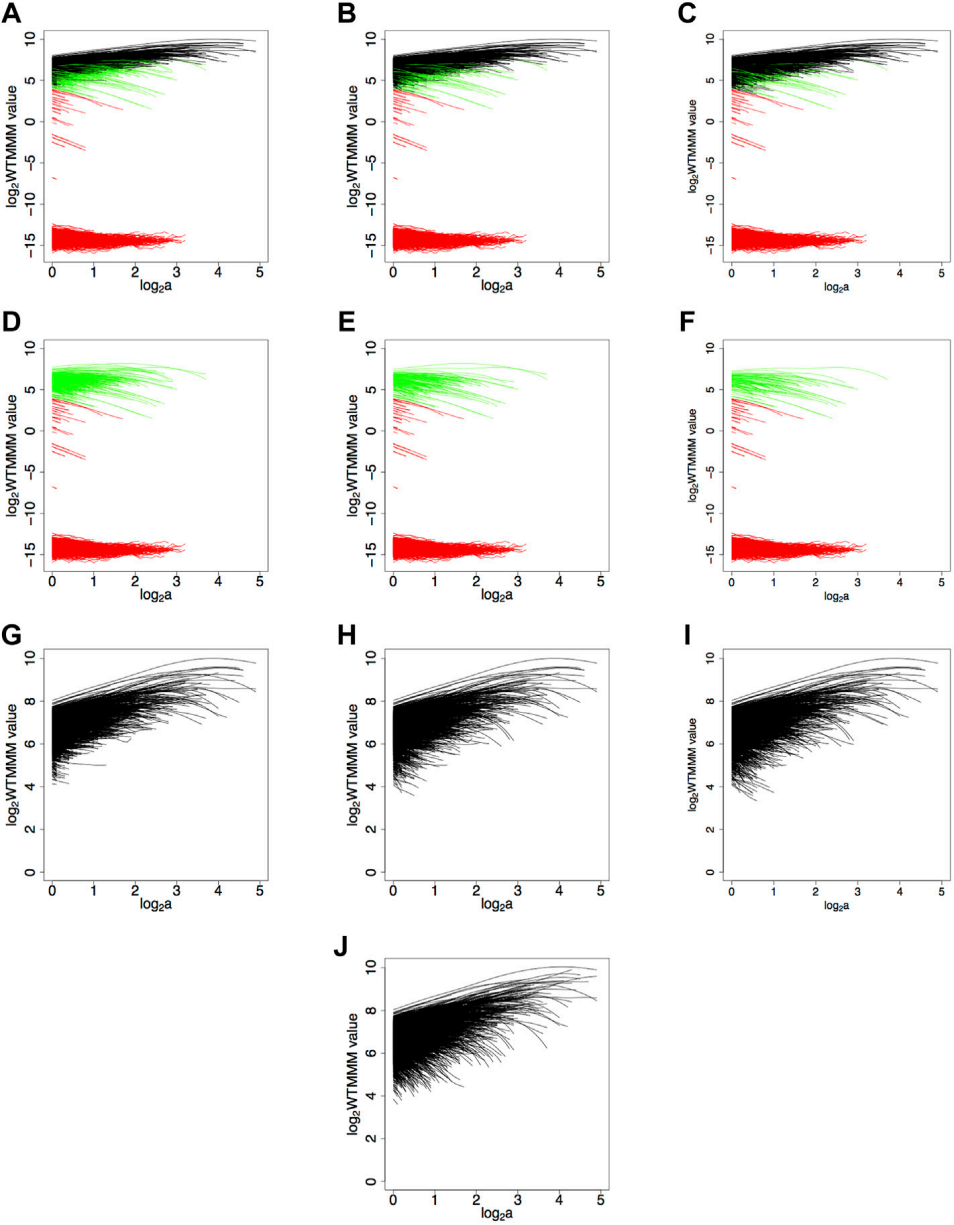
Determination of the numerical values for *SF* and *MF* for a fBm surface with *H* = 0.7 and saturation level of 20%. Maxima lines that were eliminated due to the modulus filter $\left(M_{l\left(a=1\right)}\leq MF\right)$ , are shown in red, while maxima lines that were eliminated due to the adjusted slope filter (*m*_*adj*_ ≥ *SF*) are shown in green. The remaining maxima lines are shown in black. Here a fixed modulus filter value of log_2_ (*MF*) = 4 is used while the left column has *SF* = 0.2, the middle column has *SF* = 0.5, and the right column has *SF* = 0.8. In **(A–C)** the complete original sheaf is shown with the eliminated and kept maxima lines, in **(D–F)** only the eliminated maxima lines are shown, and in **(G–I)** only the kept maxima lines are shown. As a comparison, the sheaf from the unsaturated surface is shown **(J)**.

The data mining approach that we took to determine the optimal value for *SF* is explored in Figure 7 for a fBm surface with *H* = 0.7 with 20% saturation, where a fixed value of log_2_ (*MF*) = 4 was used, with *SF* = 0.2, 0.5, 0.8 in the left (Figures 7A,D,G), center (Figures 7B,E,H), and right (Figures 7C,F,I), respectively. Our goal was to explore different values of *SF* that would remove the maxima lines that caused the saturated sheaf to look different from an unsaturated sheaf. For instance, in Figure 7G (*SF* = 0.2), the resulting filtered saturated sheaf has maxima lines that are truncated (too many good maxima lines were eliminated) while Figure 7I has many negative sloping, low valued maxima lines compared to the unsaturated case (not enough maxima lines were eliminated). Figure 7H displays what was determined to be the optimal filtering coefficients. Figure 7J is the sheaf corresponding to the unsaturated fBm surface. Note that although only three different values of *SF* and a single value of *MF* are explored in Figure 7, we numerically explored a much larger collection of values for these parameters (data not shown).

**FIGURE 8:**
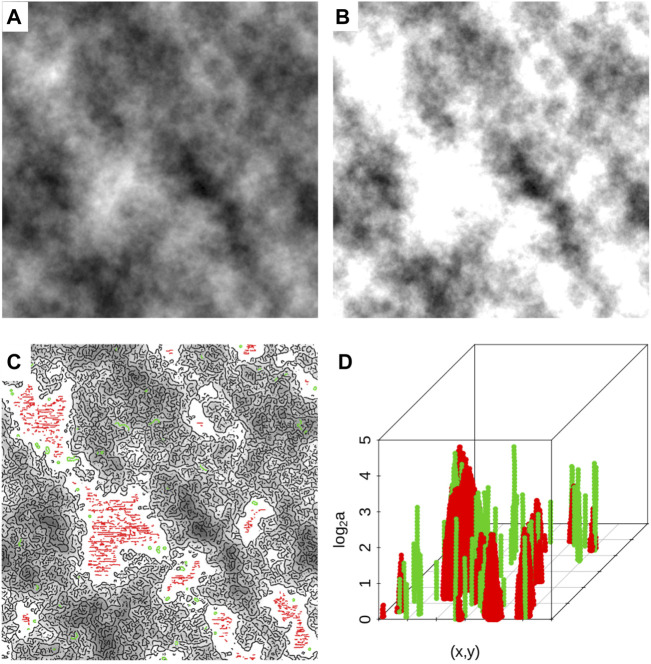
**(A)** A sample fBm surface with *H* = 0.7. **(B)** The same surface as in **(A)** but saturated at 20%. **(C)** The saturated surface overlaid with maxima chains (at scale 20 pixels) that were kept (black), those that were eliminated by applying the modulus filter log_2_ (*MF*) = 4 (red), and those eliminated by applying the slope filter *SF* = 0.5 (green). **(D)** The corresponding extraneous maxima lines that were removed.

A sample fBm surface with *H* = 0.7 at 20% saturation level is shown in Figures 8A,B. Also shown are the maxima chains, color-coded according to their elimination process (red based on the *MF* and green based on the *SF*) in Figure 8C. And Figure 8D shows the corresponding skeleton of eliminated maxima lines.

### 3.2.2 Pruned Sheafs

**FIGURE 9:**
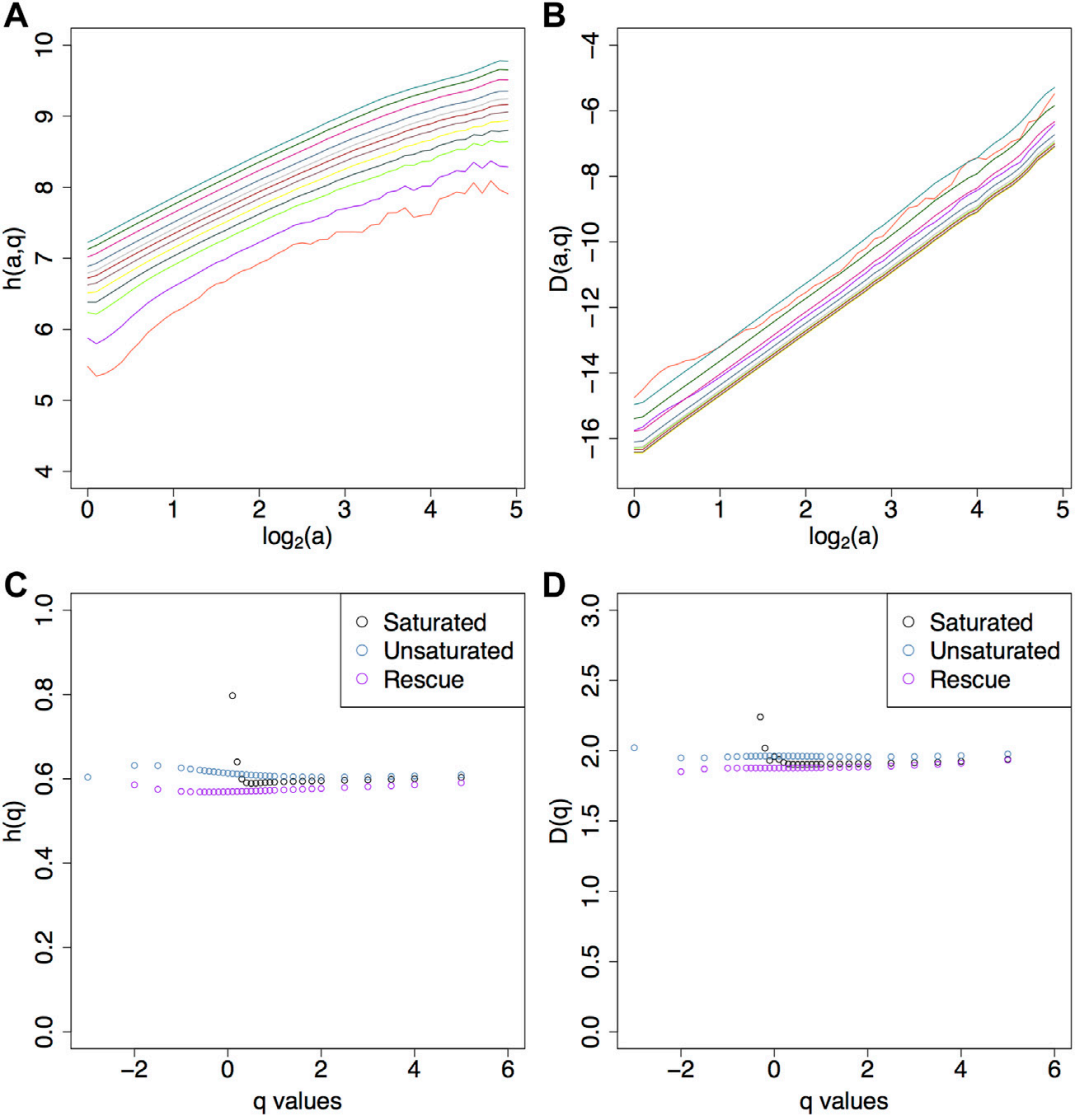
Efficacy of the rescue method. The *h* (*a*, *q*) **(A)** and *D* (*a*, *q*) **(B)** plots have better power law fits for a wider range of statistical order moments relative to their saturated counterparts. This expanded range of *q* values is reflected in the *h*(*q*) **(C)** and *D*(*q*) **(D)** plots. The saturated images had a *q* range 0.1 < *q* < 5 while the rescue method had −2 ≤ *q* ≤ 5.

Using the filtering parameters selected through the data mining approach outlined above, (log_2_ (*MF*) = 4 and *SF* = 0.5) these resulting “pruned” sheafs were then passed to the partition functions calculations (Eq. 4). From these, we get *h*(*a*,*q*) (Eq. 8) and *D*(*a*,*q*) (Eq. 9) shown in Figures 9A,B, respectively, where we can see the lack of stair step behavior compared to their saturated counterparts in Figures 6A,B and Supplementary Figures S1–S3. With these now robust *h*(*a*,*q*) and *D*(*a*,*q*) curves, from which a reliable power law fit can be obtained, we are able to expand the range of statistical order moments for the calculation of *h*(*q*) and *D*(*q*), as displayed in Figures 9C,D. For reference, the saturated images had a *q* range of ∼0<q≤5 while with the rescue method the range was expanded to −2 ≤ *q* < 5.

The low modulus maxima lines (shown in red in Figures 7 and 8) are likely responsible for the step size behavior of the *h* (*a*, *q*) curves for negative *q* value shown in Figures 6 and Supplementary Figures S1–S3. However, Table 1 shows that only eliminating these maxima lines would be insufficient to eliminate the effects of saturation. Indeed, the number of maxima lines removed depends on the saturation level, which also dictates which of the two filtering processes removes more lines. For example, for the set of fBm surfaces with *H* = 0.7 and saturation level 1%, using log_2_ (*MF*) = 4 and *SF* = 0.5, the average percentage of removed maxima lines was 0.4% due to *MF* and 4.8% due to *SF*. For saturation level 5%, these averages were 3.9% due to *MF* and 4.9% due to *SF*. For saturation level 10%, more maxima lines were removed due to *MF* (9.3%) than due to *SF* (4.9%). And for saturation level 20%, the average percentages of removed maxima lines were 21.8% due to *MF* and 4.6% due to *SF*. The percentages of removed maxima lines by filtering method for each Hurst value and for each saturation level are listed in Table 1.

**TABLE 1:** Percentages of maxima lines removed by filtering processes.

Hurst Value	Filter Value	Saturation Level			
		1%	5%	10%	20%
*H* = 0.7	*MF* = 16	0.4	3.9	9.3	21.8
	*SF* = 0.5	4.8	4.9	4.9	4.6
*H* = 0.5	*MF* = 48	0.3	1.6	4.0	10.2
	*SF* = 0.5	9.6	9.8	10.0	10.4
*H* = 0.3	*MF* = 96	0.1	0.8	1.8	4.7
	*SF* = 0.5	17.1	17.6	18.2	19.5
*H* = 0.1	*MF* = 192	0.0	0.2	0.8	2.8
	*SF* = 0.5	28.2	28.5	29.3	31.0

### 3.3 The Rescue Method on Unsaturated Surfaces

**FIGURE 10:**
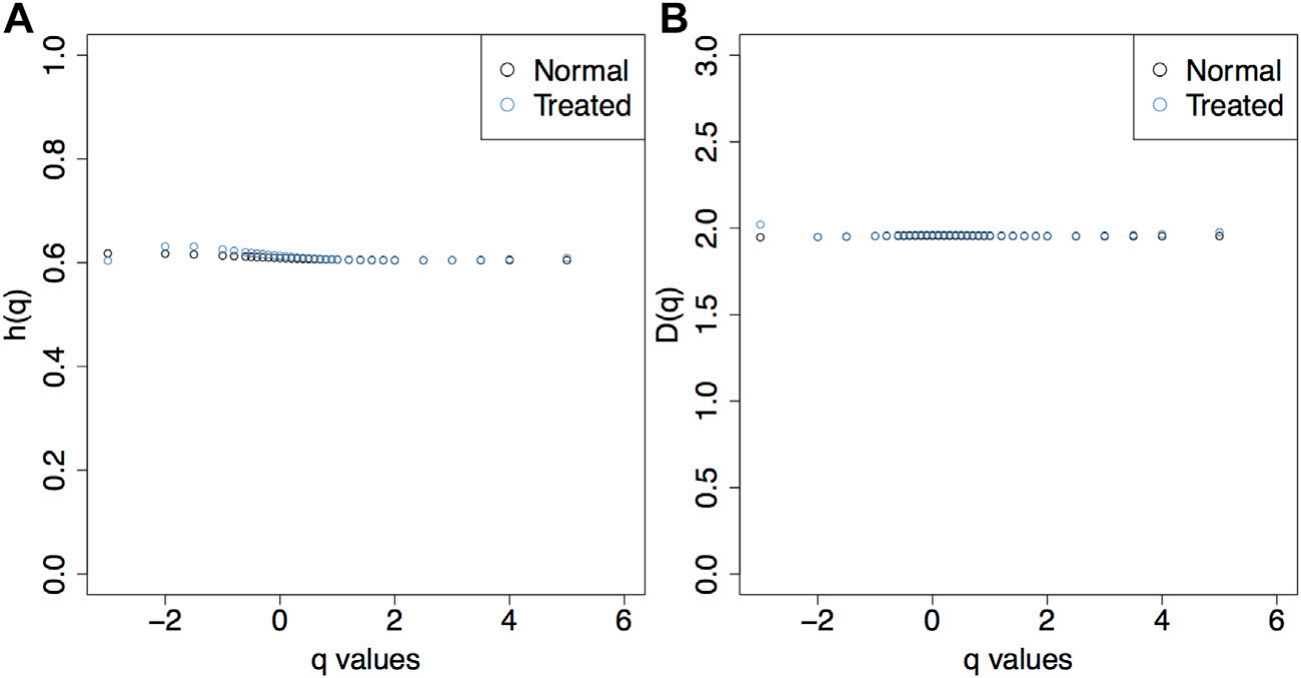
The *h*(*q*) **(A)** and *D*(*q*) **(B)** plots show that the rescue method does not fundamentally alter the behavior of the statistics when applied to unsaturated images.

We used the filtering parameters empirically determined above (log_2_ (*MF*) = 4 and *SF* = 0.5) on the sheaf of a non-saturated fBm surface with *H* = 0.7. There were only very minor differences between the *h*(*q*) and *D*(*q*) curves from the unsaturated surfaces with non-filtered sheafs (normal) vs. the unsaturated surfaces with filtered sheafs (treated) (Figure 10).

We note that the rescue method will limit the range of *q* values only if the underlying surface was saturated. This is to be expected as it is due to the removal of maxima lines from the sheaf that is fed to the partition function. Therefore, one should only compare the range of *q* values of a pruned sheaf to that of a smaller unsaturated image, for which the range of *q* values will be smaller (see Khalil et al. (2006)).

## 4 Discussion

The multifractal analysis of rough surfaces inherently assumes that these surfaces are scale-invariant and everywhere singular. These conditions are not met when image saturation regions are present. The rescue method proposed here takes full advantage of the space-scale partitioning capability that is intrinsic to the 2D WTMM multifractal method, which allows for the elimination of a subset of the wavelet transform skeleton corresponding to saturated regions.

The concept of pruning a sheaf was first introduced in Kestener et al. (2001) and then further explored in Batchelder et al. (2014). However, pruning a sheaf to mitigate image saturation effects had yet to be explored. As a contribution to the medical imaging field, data with image artifacts that would have otherwise been rejected could now potentially be rescued and included for multifractal statistical analyses.

Although several applications may benefit from this rescue method, as discussed in the introduction, an immediate benefit is for the sliding-window analysis of mammograms (Marin et al., 2017; Gerasimova-Chechkina et al., 2021). To offer the best possible computational aid to the radiologists in their interpretation of these mammograms, it is critical to extract as much quantitative information as possible from each subregion of each mammogram. A key to this success lies in reducing the number of subregions that have to be rejected, in particular due to image saturation, for which the implementation of this rescue method will be crucial.

### 4.1 Limitations and Future Explorations

In this study, only positive values of the Hurst exponent were considered for the calibration fBm surfaces (*H* = 0.1, 0.3, 0.5, 0.7), which is motivated, and justified, by the Hurst values measured in mammogram subregions (Marin et al., 2017; Gerasimova-Chechkina et al., 2021). Despite only using four Hurst values, the effects are similar, which precludes the need to investigate smaller intervals. We have no reason to suspect that exploring other values, for example *H* = 0.2, 0.4, 0.6, would produce results that require a substantially different approach. However, future efforts should be undertaken to investigate the effects of saturation on surfaces with −1 < *H* < 0. It is likely that the slope filter (*SF*) would have to be calibrated. One could also expand the investigation on multifractal surfaces (Decoster et al., 2000; Roux et al., 2000).

The numerical determination of *MF* and *SF* was done subjectively. We observe that a constant value of *SF* = 0.5 worked well for all Hurst values considered in this study, whereas *MF* is strongly correlated with the Hurst exponent. A future effort could be to implement objective approaches. For example, an automated determination of these two thresholds could be implemented based on the calculation of outliers in the probability density functions of log(M) and *m*_*adj*_.

Another improvement to the approach could be to use two thresholds instead of one for *SF* (e.g., *SF*_*high*_ and *SF*_*low*_). Maxima lines for which *m*_*adj*_ ≥ *SF*_*high*_ would be eliminated, those for which *m*_*adj*_ < *SF*_*low*_ would be kept, and those in between would require an additional classification procedure. And finally, there may be methods from machine learning to use in order to improve the maxima lines classification, assuming the unsaturated image has scale-invariant properties.

We note that the calculations of the Hurst values reported in this manuscript are underestimates of the theoretical values. This is a known phenomenon for 1D fBm signals (Muzy et al., 1991, 1994; Arneodo et al., 1995) and 2D fBm surfaces (Arneodo et al., 2000). The underestimates reported here are perhaps slightly more accentuated than previous works (Arneodo et al., 2000; Khalil et al., 2006). This could be due to the fact that a fixed universal range of scales was used for every set of fBm surfaces and all saturation levels in this study, as mentioned in Section 2.3.

The rescue method presented in this manuscript could be used on any medical imaging where the 2D WTMM multifractal method is applicable, such as lung CT scans, or histology slides (Khalil et al., 2009). It is also likely that the rescue method could be integrated into the 1D WTMM method, and therefore applicable to 1D signals such as electro-encephalograms (Richard et al., 2015) or thermography time series (Gerasimova et al., 2013, 2014), for which the MF and SF values would need to be fine-tuned for the application.

## Data Availability

The raw data supporting the conclusions of this article will be made available by the authors, without undue reservation.

## Supplementary Material

Supplementary Figure S1
**(A)** *h*(*a*, *q*), **(B)** *D*(*a*, *q*), **(C)** *h*(*q*), and **(D)** *D*(*q*) for *H* = 0.7 with 1% saturation. The colors corresponds to the different *q* values as described in the legend in Figure 4D.

Supplementary Figure S2
**(A)** *h*(*a*, *q*), **(B)** *D*(*a*, *q*), **(C)** *h*(*q*), and **(D)** *D*(*q*) for *H* = 0.7 with 5% saturation. The colors corresponds to the different *q* values as described in the legend in Figure 4D.

Supplementary Figure S3
**(A)** *h*(*a*, *q*), **(B)** *D*(*a*, *q*), **(C)** *h*(*q*), and **(D)** *D*(*q*) for *H* = 0.7 with 10% saturation. The colors corresponds to the different *q* values as described in the legend in Figure 4D.
