# Supplementary figures and images for: Elimination of Image Saturation Effects on Multifractal Statistics Using the 2D WTMM Method
Source: Front Physiol. 2022 Jun 28;13:921869. doi: 10.3389/fphys.2022.921869 (PMC9273936; doi:10.3389/fphys.2022.921869)

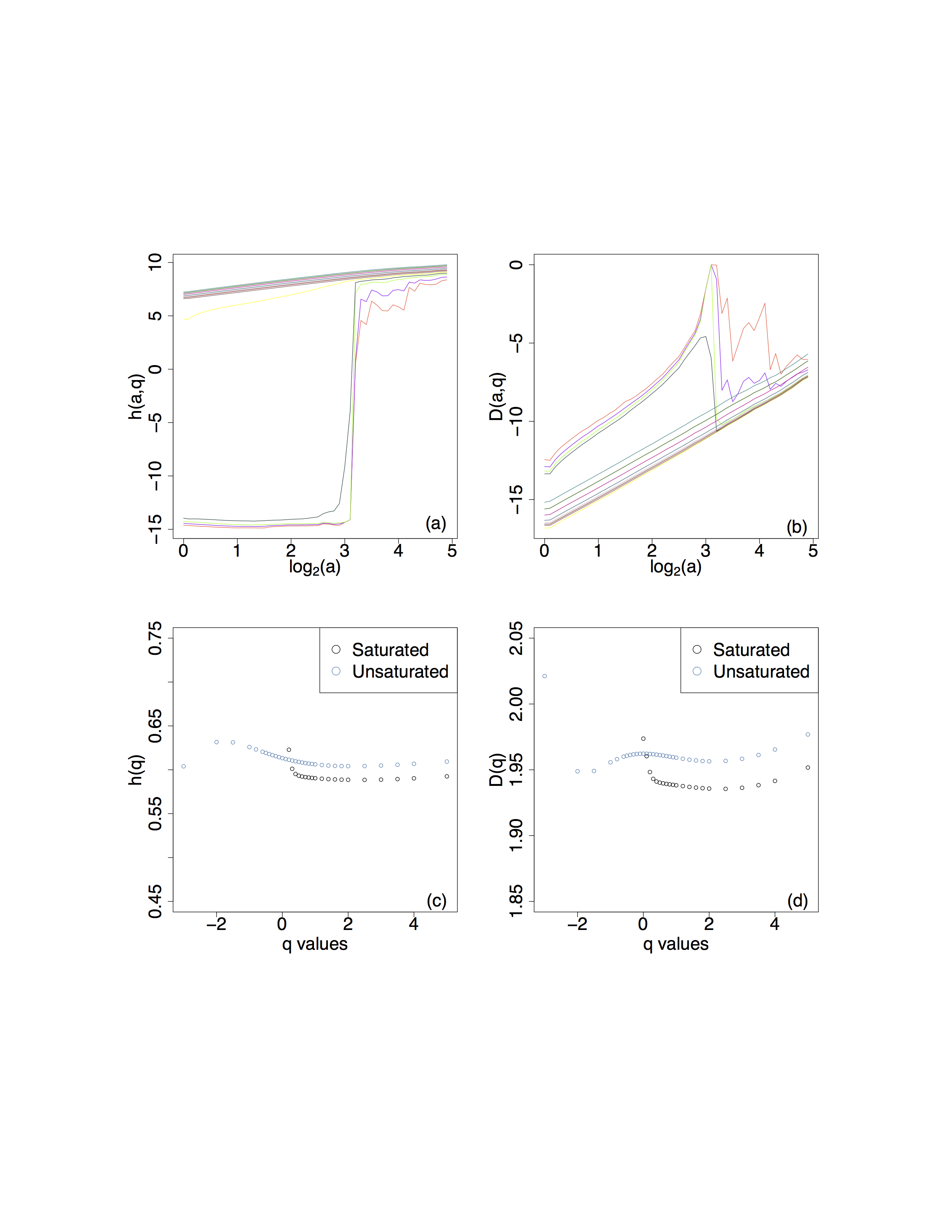

Supplement: Supplementary file 1 [file Image3.JPEG]

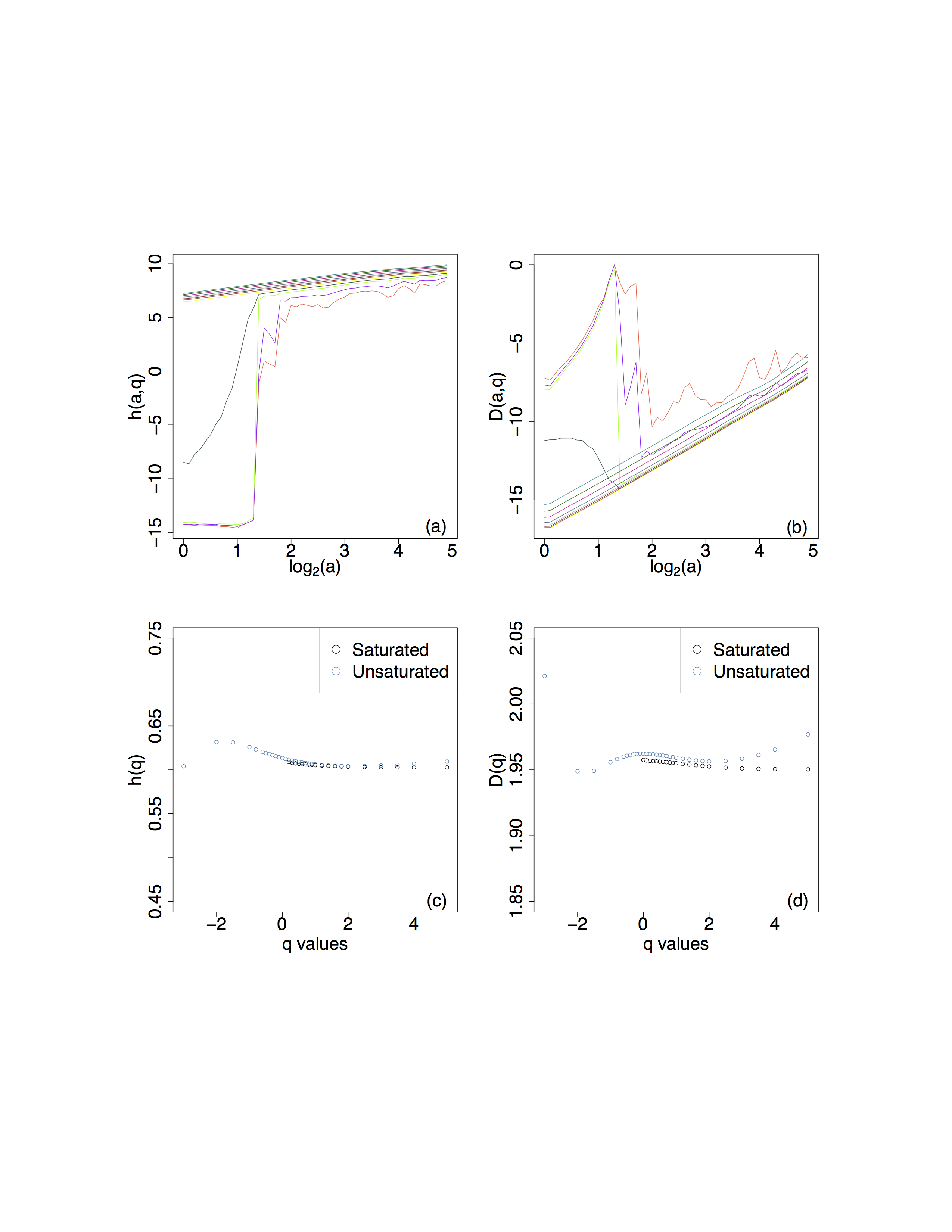

Supplement: Supplementary file 2 [file Image1.JPEG]

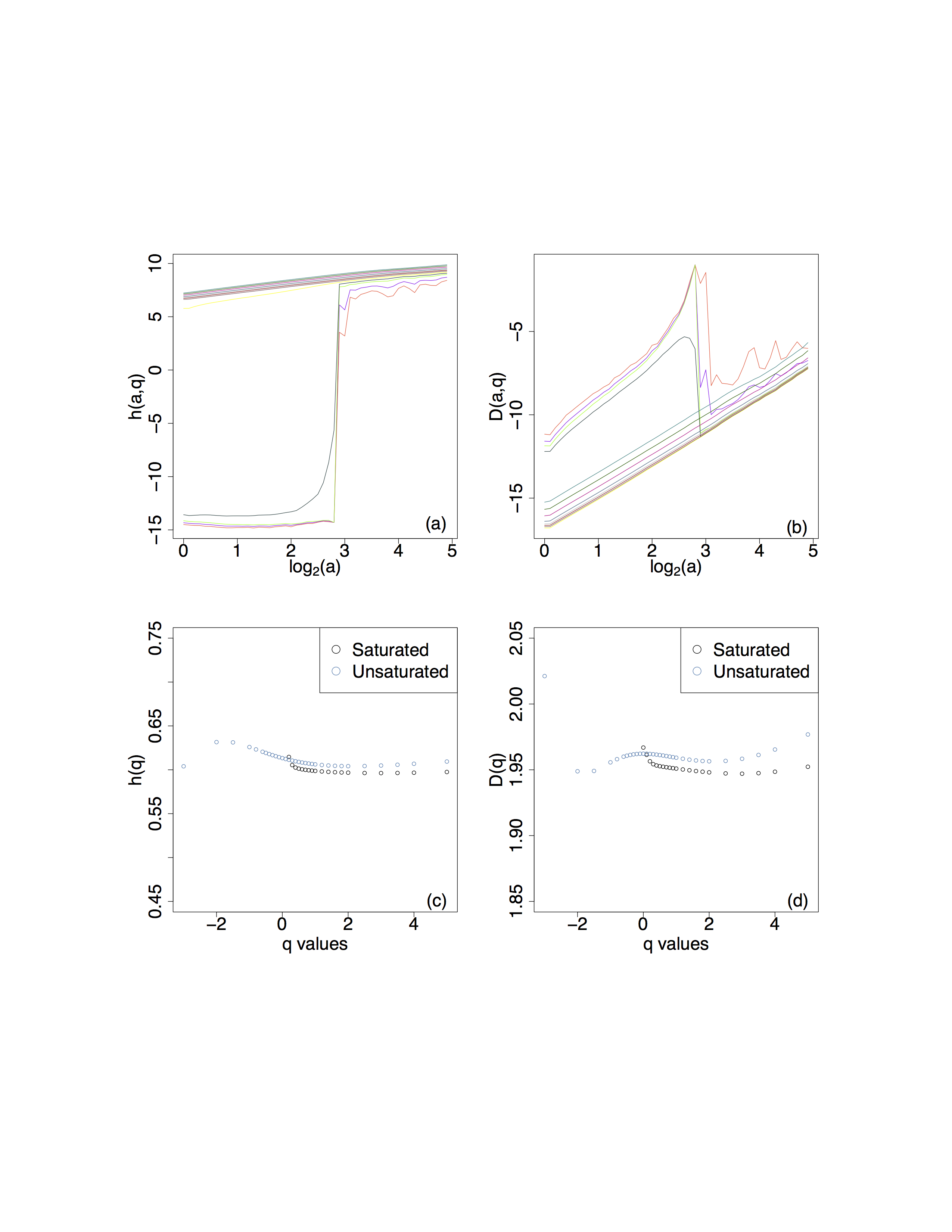

Supplement: Supplementary file 3 [file Image2.JPEG]
